# Sun exposure, ultraviolet (UV) irradiance and serum 25 hydroxycholecalciferol (25OHD) in pregnant women in rural North India

**DOI:** 10.1186/1687-9856-2015-S1-P63

**Published:** 2015-04-28

**Authors:** Siddhnath Sudhanshu, Pramod Upadhyaya, Monashish Sahu, Vinita Agarwal, Vijayalakshmi Bhatia

**Affiliations:** 1 Sanjay Gandhi Postgraduate Institute of Medical Sciences, Lucknow, Uttar Pradesh, India; 2 National Institute of Immunology, Delhi, India; 3 Max Hospital, Delhi, India; 4 Haldwani Medical College, Haldwani, Uttarakhand, India

## 

Vitamin D deficiency is rampant in India despite abundant sunshine. We aimed to estimate the amount of cutaneous vitamin D synthesis in pregnant village women (n=100) in different seasons in conjunction with serum 25OHD. We also correlated variations in surface UV energy with the presence of environmental pollution and crowding.

## Methods

The measurements of UVB radiation energy were obtained using UV spectrometer at different times of the day between 9 am and 4 pm, in different seasons. The instrument was calibrated to denote 13 microWatt/cm2 of irradiance per mVolt of deflection. Measurements were taken at our institution (situated in the countryside), at crowded inner city areas and the villages where our subjects resided. The clothing, outdoor activity pattern, and dietary calcium intake were prospectively documented. Serum 25OHD was measured by radioimmunoassay (Diasorin, Stillwater, MN).

## Results

UVB spectrometer reading ranged from 4.5 mVolts in January to 36 mVolts in June. The average erythemally effective UV energy during winter season and during the rest of the year was 308 J/m^2^ and 805 J/m^2^respectively. Average body surface area exposed was 9.5% in winter and 18.5 % in summer. Using the equation described previously by Godar et al [1] which takes into account effective erythemal irradiance, latitude, age, and duration and surface area of exposure**,** the estimated average daily cutaneous vitamin D synthesis was 769 IU during winter and 1487 IU during summer. The mean serum 25OHD was11.32 ± 5.03 ng/ml during winter (92 % < 20 ng/ml) and, 16.63 ± 8.12 ng/ml during the rest of the year (70 % < 20 ng/ml).The average peak UV irradiance calculated during April and May was significantly higher in our institute campus (338 microwatt/cm2) and the villages (312 microWatt/cm^2^), than the crowded inner city location (247 microWatt/cm^2^, p=0.03).

## Conclusion

During winter at latitude 26.8 ^0^N, cutaneous vitamin D synthesis is limited by poor UV radiation energy. Poor skin exposure is a limiting factor in all seasons. Particulate pollution may be an important remedial impediment to cutaneous vitamin D synthesis.
